# Treating schizophrenia with cariprazine: from clinical research to clinical practice. Real world experiences and recommendations from an International Panel

**DOI:** 10.1186/s12991-020-00305-3

**Published:** 2020-09-26

**Authors:** Andrea Fagiolini, José Ángel Alcalá, Thomas Aubel, Wojciech Bienkiewicz, Mats Magnus Knut Bogren, Joaquim Gago, Giancarlo Cerveri, Michael Colla, Francisco Collazos Sanchez, Alessandro Cuomo, Frieling Helge, Eduardo Iacoponi, Per-Axel Karlsson, Pradeep Peddu, Mauro Pettorruso, Henrique Jorge Ramos Pereira, Johan Sahlsten Schölin, Ingo Bernd Vernaleken

**Affiliations:** 1 School of Medicine, Department of Molecular Medicine, University of Siena, Siena, Italy; 2 Clinical Unit of Mental Health, Reina Sofia University Hospital, Cordoba, Spain; 3 Kliniken Essen-Mitte, Klinik für Psychiatrie, Psychotherapie, Psychosomatik und Suchtmedizin, Essen, Germany; 4 GGZ Keizersgracht Outpatient Mental Health Clinic, Amsterdam, The Netherlands; 5 Divsion of Psychiatry, Department of Clinical Sciences, Lund University Hospital,, Lund, Sweden; 6 Mental Health Department, Nova Medical School, Lisbon, Portugal; 7 Department of Mental Health and Addiction, ASST Lodi, Lodi, Italy; 8 Clinic and Polyclinic for Psychiatry and Psychotherapy, University of Rostock, Rostock, Germany; 9 Department of Psychiatry, Hospital Universitari Vall d’Hebron, CIBERSAM, Barcelona, Spain; 10 Department of Psychiatry and Forensic Medicine, Universitat Autònoma de Barcelona, Barcelona, Spain; 11 Department of Psychiatry, Social Psychiatry and Psychotherapy, Hannover Medical School, Hannover, Germany; 12 Lambeth Early Onset (LEO), South London & Maudsley NHS Foundation Trust and Psychosis Studies Department, Institute of Psychiatry, Psychology and Neurosciences, London, UK; 13 Forensic Services of Norrbotten County Council, Öjebyn, Sweden; 14 Psychosis Pathway Coventry and Warwickshire Partnership NHS Trust and Buckingham Medical School, Rugby, UK; 15 Department of Neuroscience, Imaging and Clinical Sciences, D’Annunzio University of Chieti, Pescara, Italy; 16 Hospital de Magalhães Lemos, Porto, Portugal; 17 Sahlgrenska University Hospital, Gothenburg, Sweden; 18 Department of Psychiatry and Psychotherapy, Fliedner Krankenhaus Neunkirchen Kreuznacher Diakonie, Neunkirchen, Germany; 19 Department of Psychiatry and Psychotherapy, RWTH Aachen University, Aachen, Germany

**Keywords:** Schizophrenia, Antipsychotics, Cariprazine, Patient subgroups, Recommendations, Negative symptoms

## Abstract

**Background:**

Management of schizophrenia is sub-optimal in many patients. Targeting negative symptoms, among the most debilitating aspects of schizophrenia, together with positive symptoms, can result in significant functional benefits and dramatically improve quality of life for patients and their carers. Cariprazine, a partial agonist of the dopamine receptors D2/D3 has demonstrated effectiveness across symptom domains in clinical trials, particularly on negative symptoms.

**Objective:**

To obtain a broader insight from clinicians with specific experience with cariprazine, on how it affects patient populations outside the clinical trial setting.

**Methods:**

The panel addressed a series of psychopharmacologic topics not comprehensively addressed by the evidence-based literature, including characteristics of patients treated, dosing and switching strategies, duration of therapy, role of concomitant medications and tolerability as well as recommendations on how to individualize cariprazine treatment for patients with schizophrenia.

**Results:**

Patients recommended for cariprazine treatment are those with first episodes of psychosis, predominant negative symptoms (maintenance/acute phase) and significant side effects (metabolic side effects, hyperprolactinemia, sedation) with other antipsychotics. When the long-term treatment of a lifetime illness is adequately weighted, cariprazine becomes one of the first-line medications, not only for patients with predominant negative symptoms but also for those with relatively severe positive symptoms, especially if they are at the first episodes and if a specific medication is added for symptoms such as agitation or insomnia. For instance, patients with agitation may also benefit from the combination of cariprazine and a benzodiazepine or another sedating agent. Cariprazine may be prescribed as add-on to medications such as clozapine, when that medication alone is ineffective for negative symptoms, and sometimes the first may be discontinued or its dose lowered, after a period of stability, leaving the patient on a better tolerated antipsychotic regimen.

**Conclusions:**

Based on real-world clinical experience, the panel considered that cariprazine, with its distinct advantages including pharmacokinetics/pharmacodynamics, good efficacy and tolerability, represents a drug of choice in the long-term management of schizophrenia not only for patients with predominant negative symptoms but also for those with positive symptoms.

## Introduction

What do King Henry VI and the Nobel laureate John Nash have in common? Both were thought to have had schizophrenia. While the term schizophrenia is less than 100 years, it was first identified as a discrete mental illness in 1887 and is believed to have accompanied mankind through its history [1, 2]. Schizophrenia is a complex heterogenous, stigmatised mental disorder with multiple domains of presentation and a highly variable clinical course that can devastate individuals and families. It affects approximately 1% of the general population with an estimated 20 million cases worldwide [3, 4]. Treatment modalities in schizophrenia have changed fundamentally in recent decades and fortunately the days of people with the disease being confined to treatment in asylums are far behind us. We now have a range of antipsychotic agents and rehabilitation strategies with proven efficacy in treating symptoms and behaviours associated with the disorder [5]. However, there are still treatment gaps—with one source indicating that only about half of all patients with schizophrenia receive treatment [6].

For instance, the clinical manifestations of schizophrenia are heterogeneous and this makes it difficult to establish the effectiveness of specific treatments in a group of patients presenting with quite diverse clinical manifestations. Also, more than half of patients with schizophrenia experience negative symptoms, which are connected with significant treatment challenges [7]. In fact, negative symptoms have been associated to worse treatment response and poor functional outcomes compared with individuals without negative symptoms [8].

Domains of presentation in schizophrenia include positive, negative and emotional symptoms as well as cognitive and social functional deficits. Generally, antipsychotic agents are most effective in treating the positive symptoms of schizophrenia and less successful in treating the negative symptoms and cognitive disabilities. Targeting negative symptoms (apathy, lack of emotion, alogia, anhedonia, and poor social functioning) can result in significant functional benefits and dramatically improve quality of life of patients and their carers [9]. Indeed, the evidence underscoring the importance of negative symptoms’ resolution has quickly grown, paralleling the growing interest in functional recovery and the observation that resolution of positive symptoms is not sufficient alone to lead to functional recovery, with full functional/social recovery occurring in 15% or less of patients with schizophrenia, with negative symptoms playing a key role [9].

Despite the complexity and heterogeneity of schizophrenia, the reduction of D2-like receptor mediated neurotransmission was regarded to be the fundamental mechanism of antipsychotic action. However, recent studies have proposed that alteration in the glutamate, GABA, acetylcholine, and serotonin system may play a role in the pathology of schizophrenia and a revised dopamine hypothesis suggests a role of both hyperactive dopamine transmission in the mesolimbic areas and hypoactive dopamine transmission in the prefrontal cortex [10]. To this end partial agonism as well as the differentiated recognition of the D2-like dopamine receptors provide a promising approach for treatment of schizophrenia [11]. Cariprazine, a partial agonist of the dopamine receptors receptor and—with higher affinity—of the D3-receptor, was approved by the US Food and Drug Administration and the European Medicines Agency, for the management of schizophrenia based on an extensive clinical trial programme demonstrating its effectiveness particularly on negative symptoms and its safety [12, 13]. Since its approval the exposure to cariprazine is around 50,000 patient/treatment/year [14].

Cariprazine is pharmacodynamically and pharmacokinetically distinct from other oral antipsychotics in that it has two active metabolites which contribute to its efficacy and tolerability—the parent drug and the desmethyl cariprazine (DCAR) metabolite are responsible for early efficacy and side effects, while the second metabolite didesmethyl cariprazine (DDCAR) with its extended half-life is primarily responsible for later efficacy and tolerability issues [15–17].

Optimal management requires accurate patient characterization and the heterogeneity of schizophrenia means that subjects included in clinical trials may not be entirely representative of real-world patients [18]. We now know that clinical trial data alone may not accurately reflect how a drug will behave long-term in a larger, less homogenous patient population. Real-world experience provides a broader insight into the how a product will affect patient populations outside the clinical trial setting. With this in mind, a group of European psychiatrists (chosen from a range of EU countries, where health systems and prescribing habits vary widely), met in December 2019 to present and discuss their clinical experience with cariprazine.

Our hypothesis was that specific treatment algorithms could be suggested based on the characteristics of patients treated, for instance to establish dosing and switching strategies, duration of therapy, role and type of concomitant medications and strategies to improve tolerability. We also hypothesized that it was possible to develop a consensus on the recommendations to individualize cariprazine treatment for patients with schizophrenia.

This statement represents the panel’s collective experience, opinions, evaluation, and rationale for the inclusion of cariprazine in the management algorithm for medication treatment of schizophrenia.

## Methodology

This was a descriptive survey of a panel of clinicians and researchers with extensive experience in the management of schizophrenia in general and the use of cariprazine in particular. The panel focused on a series of psychopharmacologic topics not comprehensively addressed by the evidence-based literature but beneficial to clinicians prescribing antipsychotic medications and caring for patients with schizophrenia. The process took the form of the following steps. First, a group of clinicians and clinical researchers practicing in Europe or in the United Kingdom were surveyed about their ability and willingness to provide clinical experience, scientific background and decision-making authority to supply real-world experience with cariprazine. Second, one of the clinicians (AF) drafted a list of clinically relevant topics and questions pertaining to the treatment of schizophrenia with cariprazine in the real world. Third, the panel participants met in person and were asked to discuss the topics above and offered to add other question or topic that they felt was clinically relevant for real-world treatment of schizophrenia with cariprazine. The final survey, included the following topics: (1) place in therapy and clinical characteristics of patient populations treated with cariprazine by panel members in their respective clinical centres (both in- and out-patient facilities); (2) most frequently used doses of cariprazine in their practice; (3) procedures used in switching from other antipsychotics to cariprazine, up-titration and time to observation of beneficial effects: acute/maintenance treatment; (4) use of concomitant medications and duration of therapy by patient group/setting; (5) combination treatment; (6) nature, incidence and management of adverse events and the role of treatment adjustment/withdrawal; and (7) recommendations to individualize cariprazine treatment for patients with schizophrenia. Each of the thematically defined discussions was supplemented by systematically evaluations and ratings of the proposed topics. A number of the questions included Likert scales (0–10) and free text replies to measure opinions, perceptions, and behaviours. Clinicians completed questionnaires electronically, during the in person meeting, and answers were stored remotely for analysis. All completed answers were electronically and anonymously recorded during the meeting, and no demographic data were collected.

## Results: Data gathering and recommendations

### Cariprazine: place in therapy, patient characteristics

What are the most frequent clinical conditions for which you use cariprazine in your clinic?

The panel members indicated use of cariprazine in patients with a wide spectrum of presenting symptoms or conditions (Table 1). Cariprazine was most often prescribed for patients presenting with their first episode of psychosis—in general these tend to be younger patients; sufficient clinical experience with cariprazine in elderly patients (over 65 years) was not reported. The two other major groups considered to be ‘first-line candidates’ for cariprazine are those with predominantly negative symptoms and those with metabolic syndrome including ethnic groups/non-caucasians with diverse metabolic profiles.

**Table 1:** Spectrum of presenting conditions for which Panel prescribed cariprazine in their practice (in- and out-patients)

First episode of psychosis
Positive symptoms
Negative symptoms
Patients with schizophrenia spectrum disorders and agitation (in combination with a second medication, e.g., a benzodiazepine)
Patients with schizophrenia spectrum disorders and metabolic syndrome
Patients with schizophrenia spectrum disorders and substance use disorder

### Most frequently used doses of cariprazine in clinical practice

**Fig. 1:**
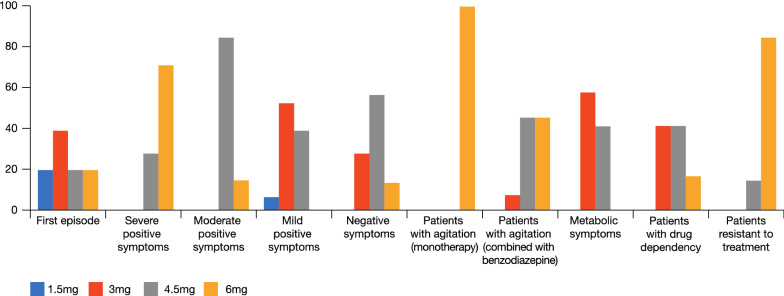
Cariprazine dosages used by Panel (%) to treat schizophrenia in their clinical practice

What are the most frequently used doses of cariprazine in your clinic? For patients with a first episode of psychosis the general consensus was that the majority of patients can be successfully managed on 1.5–3.0 mg but this depends on several issues, such as severity of symptoms and setting of treatment (community/in patients), with more severe symptoms often requiring higher doses, and other patient characteristics, for example BMI (higher BMI usually requires longer periods to achieve steady state and hence therapeutical levels and may require a faster titration schedule or higher initial dose, to attain an earlier achievement of the therapeutical blood concentrations). When severe symptoms are present, there is usually a need to quickly achieve a higher dose (4.5/6 mg). The panel judged that patients with agitation who are treated with cariprazine very often need adjunctive treatment with a benzodiazepine or another sedating agent (See: “Use of concomitant medication and duration of therapy by patient group/setting”—patients with insomnia or agitation). In those cases, cariprazine is usually needed at doses of 4.5–6 mg. If side effects such as akathisia appear, the dosage should be reduced (See: “Nature, incidence and management of side effects and the role of treatment adjustment/withdrawal” section). When cariprazine is used to treat a patient with schizophrenia and acute agitation, the advantage is that it can be continued long-term thus avoiding the need to start with a sedative antipsychotic drug and then switch treatment after the acute phase has passed. The majority of the panel considered that treatment-resistant patients require the highest dosage (6.0 mg). A large portion of physicians reported improvements in symptoms with 3.0 mg, in particular favourable long-term effects on negative symptoms with 3.0 mg without the need to increase the dosage. The possibility of using therapeutic drug monitoring (TDM) for dose optimization and to respond to individual differences during the course of therapy were mentioned. Figure 1 reports cariprazine dosages used by Panel to treat schizophrenia in their clinical practice (Fig. 1).

### Procedures used in switching from other antipsychotics to cariprazine, up-titration and time to observation of beneficial effects: acute/maintenance treatment

The overriding consideration in switching stable patients who need to change medication because of side effects, or to better deal with residual negative symptoms/cognitive disabilities, is to avoid relapse and the panel were in agreement of the need to ensure that an effective dose of cariprazine is reached before tapering. In some cases, the risk of increased side effects is offset by avoiding relapse, but it is important to obtain a balance. The long-half life of cariprazine gave reason to the opinion that it can be up-titrated quickly followed by final adjustments of daily dose, while blood levels gradually increase, up to steady state according to previous pharmacokinetic investigations. After multiple dose administration mean cariprazine and DCAR concentrations reached steady state at around weeks 1–2 and mean DDCAR levels approached steady state at around weeks 4–8, in a 12-week study [16]. Using the half-life of total cariprazine (the sum of cariprazine and its major active metabolites DCAR, and DDCAR) a time marker of 3 weeks for a steady state has been estimated. Population pharmacokinetic/pharmacodynamic modelling has been used to investigate the longitudinal exposure‐response relationship for total cariprazine in patients enrolled in the clinical development program. Total cariprazine exposure was significantly related to reductions in Positive and Negative Syndrome Scale (PANSS) total scores and typical steady‐state plasma concentrations after 3.0 and 4.5 mg/day were associated with 50% of maximum typical reductions in PANSS scores [17].

Clinicians reported that they usually introduce cariprazine while slowly reducing other antipsychotics (if taking). The length of time in switching depends on the type of drug(s) the patient has already been prescribed and TDM may be useful for dose optimization. In the panel’s experience switching from the partial agonist aripiprazole can be done in 1 week or less but a longer overlapping time is necessary when switching from medications such as risperidone or haloperidol. Care must be taken when down-titrating medications with pronounced antihistaminergic/antimuscarinic effects like olanzapine, quetiapine or clozapine. In stable patients, switching from risperidone is not usually associated with histaminergic/muscarinic rebound. However, a dopaminergic rebound is possible and hence an overlap of at least 2–3 weeks is usually recommended. If the patient is stable and treated with olanzapine, quetiapine or clozapine, the switch will be usually performed over a longer time period (3–4 weeks) owing to the risk of both antihistaminergic/muscarinic and dopaminergic rebound. Overlapping ‘plateau’ switch strategies (the effective blood concentration of the new drug is reached before reducing/stopping the other one) and transient co-treatment with medications, such as benzodiazepines (e.g., lorazepam 1-4 mg/day) are used to overcome the rebound phenomena that can complicate the early switch period. The time employed in up-titration of cariprazine depends to some extent on the setting and practical issues—in the acute setting it is usually performed faster than in outpatient clinics according to the frequency of visitation as well as due to different urgencies of the clinical situations (Table 2). With outpatients who may be seen less frequently, it takes longer to up-titrate/switch with medications with long-half life. A portion of patients, around 25%, experience akathisia when switching to cariprazine which decreases the likelihood of a successful switch-as patients are reluctant to continue. In these cases, the dosage is increased in a stepwise fashion at longer (e.g., 2-week) intervals. This takes longer but improves efficacy and adherence in the long-term (See: “Nature, incidence and management of side effects and the role of treatment adjustment/withdrawal” section). The time to observe beneficial effects depends on the patient and the dosage used: improvements are observed in around 1 month for positive symptoms, but complex negative symptoms take up to 6 months and longer to improve.

**Table 2:** Up-titration with cariprazine in acute and maintenance settings

Fast Acute	Slow Maintenance
In acute setting up-titrate to 4.5 mg or 6.0 mg in 1 week—increase 1.5 mg every day up to 6.0 mg if no side effects manifest	Up-titration should be slow in maintenance, whereas fast titration is not necessary in maintenance
Fast titration appropriate for hospital setting due to the need to shorten the length of stay in the hospital	Stopping in case of side effects 1 week if replaced with another neuroleptic. Reducing dose in case of beginning of metabolic problems, stop immediately in case of worsening psychotic feature (positive symptoms)
Side effects may delay up-titration, but goal is to reach effective dose as soon as possible	The slower the better if side effects such as akathisia manifest
When used as monotherapy in highly ‘acute’ cases fast up-titration required	If body weight is over 120 kg should start with the 3.0 mg dosage
Switch 1.5 mg every 3 days is acceptable and usually less likely to produce akathisia	Vulnerable patients must be titrated much slower

### Use of concomitant medications and duration of therapy by patient group/setting

#### Patients with insomnia or agitation

**Fig. 2:**
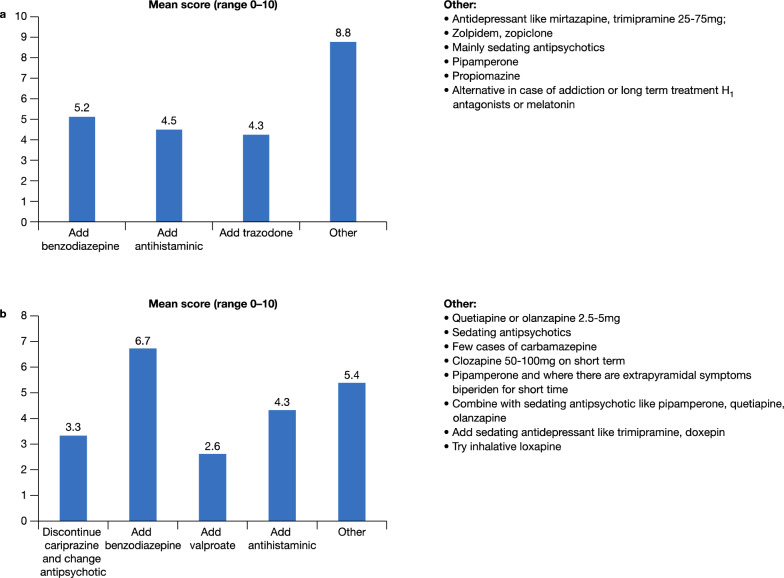
Medications used by Panel to treat concomitant insomnia (**a**) and agitation (**b**)

A cross section of responders would prescribe a benzodiazepine to treat insomnia but for some the use of a benzodiazepine depends on the setting and the risk of addiction and other side effects, such as paradoxical activation, sedation, falls, and withdrawal symptoms if the medication is discontinued too quickly (Fig. 2a). Antidepressants, such as mirtazapine or trazodone, or sedating antipsychotics at low doses, may be alternatives to a benzodiazepine. Similarly, to manage concomitant agitation the group were distributed as to add a benzodiazepine (which had the highest score) or a second sedating antipsychotic/antidepressant agent but not at antipsychotic doses, or to avoid using two antipsychotics, i.e., discontinue cariprazine and change antipsychotic (Fig. 2b).

#### Partial response to positive or negative symptoms

**Fig. 3:**
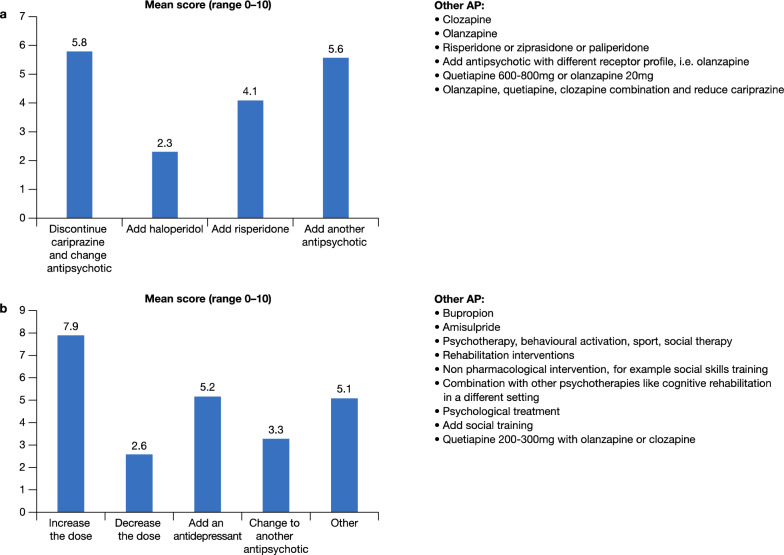
Strategies employed when there is a partial response for positive symptoms (**a**) and negative symptoms (**b**)

In patients showing partial responses to positive symptoms after treatment with cariprazine for a period of 2–3 weeks, one option is to increase the dose of cariprazine to the highest dosage. If this is not adequate another antipsychotic is added (Fig. 3a). Similarly, if there is a partial response for negative symptoms—the dose can be increased and/or add an antidepressant and/or add/switch to another antipsychotic (Fig. 3b). The role of non-pharmacological therapies should, however, not be underestimated—if there is a partial response for negative symptoms then the patient can be motivated to start psychotherapies or rehabilitation interventions such as cognitive rehabilitation and social skills training, which then can have a synergistic/additive effect.

### Combination therapy

**Fig. 4:**
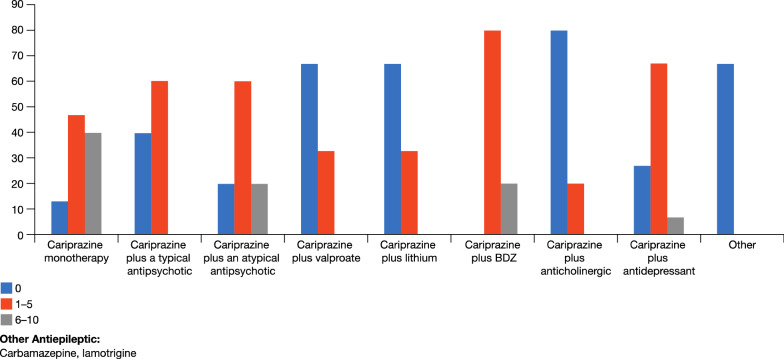
Frequency (%) of cariprazine as monotherapy and in combination

The panel reported that cariprazine monotherapy is indicated for patients with schizophrenia spectrum disorders. However, for partial responses and patients with comorbidities (schizoaffective disorder, agitation, aggression, substance abuse) combinations may be necessary (Fig. 4). It is important to ensure drug interactions do not occur to avoid side effects such as dizziness, drowsiness, confusion, and difficulty concentrating. Also, it is not advisable to use carbamazepine, or another CYP3A4 inducer, in combination with cariprazine; moreover, clinicians should evaluate very carefully adding valproic acid in females, due to the teratogenic risk. Cariprazine can be given in combination with lithium in patients at risk of suicide.

### Nature, incidence and management of side effects and the role of treatment adjustment/withdrawal

**Fig. 5:**
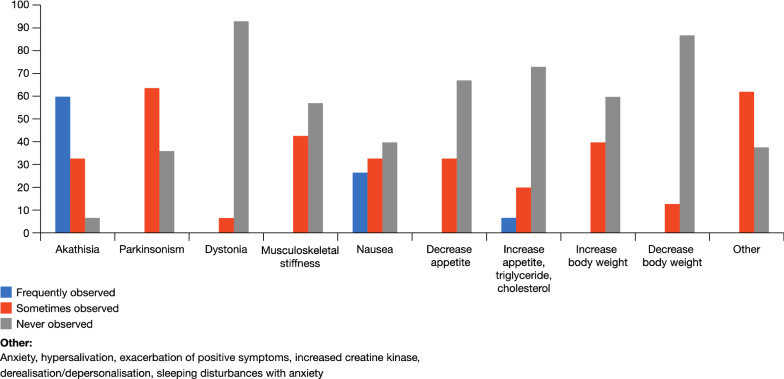
Side effects (%) most commonly observed in physicians’ everyday clinical practice

The most common and clinically bothersome adverse event with cariprazine is akathisia (Fig. 5). Increase in body weight, metabolic syndromes and cardiovascular changes are not reported to be particularly problematic. In many cases, when cariprazine is discontinued it is not due to lack of efficacy but adverse events which are patient dependent. In most cases adverse events are successfully managed by dosage reductions or, in the case of acute akathisia, short-term combination with a benzodiazepine or beta-blocker.

### Recommendations to individualize cariprazine treatment for patients with schizophrenia

Due to the specific pharmacodynamics profile, (partial agonist of the dopamine receptors D2/D3, with a tenfold affinity for the D3 receptor with partial agonism for the serotonin [5-hydroxytryptamine] 5HT1A receptor, as well as antagonism at 5HT2B and 5HT2A receptors and for the histamine H1 receptor), the panel members judge cariprazine to be a drug with important clinical and pharmacological advantages over other antipsychotic agents [17, 19–21]. The main advantage is assumed in its superior efficacy in treating the negative symptoms of schizophrenia—representing a major step forward for patients, carers and healthcare professionals alike [22, 23]. The panel noted the evidence on the ability of cariprazine to reduce troublesome side-effects that frequently cause patients to discontinue some of the other antipsychotics, including anti-cholinergic (dry mouth, constipation, urinary retention, exacerbation of the dangerous effects of closed-angle glaucoma), anti-adrenergic (orthostatic hypotension), antihistaminergic (sedation, weight gain) and metabolic (weight gain, increased cholesterol, increased triglycerides) as well as a reduced risk of arrhythmias [24, 25].

**Fig. 6:**
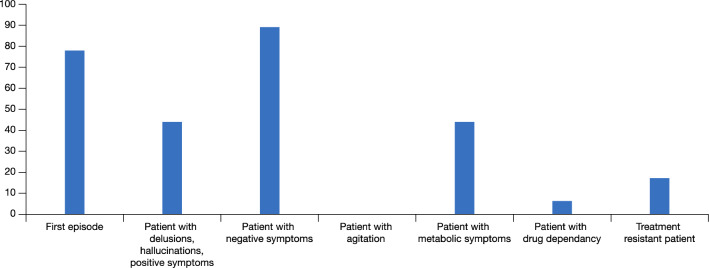
Patients groups the Panel considered would benefit treatment with cariprazine

The panel recommended patients with negative symptoms—which are among the most debilitating aspects of schizophrenia—as a target group for cariprazine (Fig. 6) [26]. The importance of preparing a long-term treatment plan is sometimes lost sight of, in particular when relevant acute positive symptoms triggered the initial treatment decisions. If the initial antipsychotic strategy does not significantly improve negative symptoms, a medication switch may be necessary once the positive symptoms are treated. In many situations healthcare professionals concentrate on the acute phase of schizophrenia (psychoses), because it is the most ‘urgent’ but the resolution of negative symptoms or of side effects is just being postponed. Furthermore, sedation is a major issue in the management of schizophrenia—it may be welcomed in the acute phase but is detrimental in the long-term as it prevents patients from functioning and focusing on the future and in some cases causes patients to be non-adherent. Schizophrenia patients with metabolic issues are dying 15 years earlier, inter alia, due to metabolic issues so a drug such as cariprazine with a low level of metabolic problems—there has been a recent report of a reversal of the metabolic syndrome with cariprazine—represents a major advance in the management of this patient group [27].

When cariprazine is administered at the outset to first psychotic episode patients (acute/in-patients/in the community) to control both positive and negative symptoms, the need for a switch once positive symptoms are controlled might be lower (Fig. 6).

Another patient group in whom the panel considered cariprazine can make a difference is in patients with schizophrenia and concomitant substance use disorder. A large portion of the patients the panel see with schizophrenia also consume illicit drugs and there are limited treatment choices that can make a difference. If a patient continues to, for example, smoke cannabis, is non-adherent or partially adherent and does not want to take a depot formulation, the long-half life and non-sedative action of cariprazine make it the drug of choice. Cravings are a part of the ongoing cycle of addiction and certain agents can prevent cravings (illicit drugs/alcohol) by blocking the receptors (e g., NMDA-, opioid- and dopamine receptors) associated with cues that set off relapse—cariprazine is a partial agonist at dopamine D2/D3 receptors and as such may have a role in reducing cravings in schizophrenia patients with substance use disorder [28–30]. In an animal study, cariprazine outperformed aripiprazole in decreasing the rewarding effect of cocaine and avoiding relapse after a period of withdrawal from cocaine and related cues [31]. Patients with mild or moderate agitation may benefit from cariprazine, provided that a second agent (such as lorazepam) is added. The panel considered that cariprazine is not a first choice for patients with severe agitation (Fig. 6).

Combinations of antipsychotics are commonly used in refractory schizophrenia when a single agent does not relieve symptoms adequately, but there is limited evidence on the benefits of this strategy. Cariprazine improves social functioning and as such may have a place as an add-on therapy (at low dosages) in treatment-resistant patients, including those on clozapine or on long acting antipsychotics (LAI). To our knowledge, no randomized controlled trials testing the combination of cariprazine and clozapine or cariprazine and LAI have been conducted to date. Indeed, the results from meta-analyses of antipsychotic combinations indicate that data are insufficient and that more studies are needed. However, a recent paper by Guinart and Correl, evaluated the existing evidence and concluded that ‘evidence for superior efficacy vs antipsychotic monotherapy is scant (possibly with the exception of reductions in negative symptoms when combining a partial D2 agonist and a D2 antagonist)’ [32]. Hence, the combination clozapine–cariprazine or LAI cariprazine is of interest, especially for cases of patients with treatment-resistant schizophrenia that do not respond to more supported treatments [32–34].

## Conclusions

According to the panel’s collective experience and opinions, the ideal cariprazine patients are individuals at their first episodes of psychosis, those with predominant negative symptoms during the maintenance period or during the acute phase, and those with significant side effects (i.e., metabolic side effects, hyperprolactinemia, or sedation) from other antipsychotics during periods of stability. Cariprazine may also be a drug of choice for patients with predominant positive symptoms during the acute phase, especially when clinicians consider the longer term benefits. When schizophrenia is seen as a life-long disorder, characterized by relatively brief periods of exacerbation and relatively longer periods of remission, the goal of treating acute symptoms remains paramount. However, in these cases, the efficacy in treating negative symptoms and the tolerability profile become as important as the need to treat and prevent the positive and most acute symptoms. When the long-term treatment of a lifetime illness is adequately weighted, cariprazine becomes one of the first-line medications, not only for first episode patients, patients with predominant negative symptoms and those with significant side effects from other antipsychotics but also for patients with relatively severe positive symptoms. When positive symptoms are very severe, adjunctive medications may be necessary, at least for the acute treatment period. In fact, the favourable effectiveness during the maintenance period may drive the choice in the acute phase, even at the price of using a second medication. For instance, in a patient with moderate agitation, a clinician looking exclusively at the acute phase would likely choose a sedating antipsychotic, such as olanzapine, that successfully treats all the symptoms discussed above in most patients. However, a clinician that looks at the maintenance treatment as well, may rather choose a medication like cariprazine augmented with a benzodiazepine or another medication that is effective for agitation. In fact, cariprazine is usually better tolerated than olanzapine, primarily in terms of metabolic side effects, and the benzodiazepine can be discontinued afterwards, when the control of agitation is no longer necessary.

The same may be true when a clinician chooses to augment clozapine with an antipsychotic such as cariprazine, when a patient is experiencing inefficacy of clozapine for negative symptoms. In some of these cases, once the patient is stabilised, it is then possible to decrease the clozapine dose. In a smaller yet significant number of cases, it may even be possible to completely discontinue clozapine, after a long enough period of stability, leaving the patient on a better tolerated antipsychotic. Likewise, when a patient experiences psychotic symptoms in the acute phase that are partially resistant to cariprazine, a clinician can choose to use it in combination with clozapine or risperidone.

Based on their real-world clinical experience the panel considered that cariprazine, with its distinct advantages including pharmacokinetics, pharmacodynamics, efficacy and tolerability, represents one of the drugs of choice in the long-term management of schizophrenia not only for patients with predominant negative symptoms but also for those with positive symptoms. When positive symptoms are severe and partially responding to cariprazine, the combination of cariprazine with a second antipsychotic may be considered, with the goal to attempt monotherapy once the acute phase has solved and the patient progresses to maintenance treatment.

## Data Availability

Relevant data are available on reasonable request.
